# The “Reading the Mind in Films” Task: A Pilot Study on Complex Emotion and Mental State Recognition for the Italian Adaptation in Adults with and Without Autism Spectrum Conditions

**DOI:** 10.3390/brainsci14121240

**Published:** 2024-12-11

**Authors:** Raffaele Simone Scuotto, Sofia Bonfanti, Paola Ricciardelli

**Affiliations:** Department of Psychology, University of Milano-Bicocca, 20126 Milan, Italy; raffaele.scuotto@unimib.it (R.S.S.); s.bonfanti27@campus.unimib.it (S.B.)

**Keywords:** autism spectrum condition (ASC), emotion recognition, reading the mind in film (RMF), reading the mind in the eyes test (RMET)

## Abstract

**Background/Objectives:** The present pilot study tested and reports the Italian adaptation of the Reading the Mind in Film test (RMF), an ecological test for assessing, in Italian adults with and without Autism Spectrum Condition (ASC), complex emotion and mental state recognition in natural settings and everyday situations. **Method**: A sample of young adults with Autism Spectrum Condition (with ASC; *n* = 22), attending a filmmaking course at a post-diploma school (*Scuola Futuro Lavoro*) took part in the study and was compared with a control group of neurotypical university students (without ASC; *n* = 22). All participants underwent individual testing and completed the Italian version of the Autism Questionnaire before performing the Italian version of both the RMF task and the Reading the Mind in the Eyes Test (RMET). The latter, widely used to evaluate the ability to detect what someone else is thinking or feeling from the eye region. **Results:** The findings of the control group were in line with the original study, demonstrating the validity and reliability of the translation and the dubbing procedure of the RMF test. However, no main significant differences in performance were found between the two groups. **Conclusions**: Such results suggest that taking a course in film and video making may have helped the autistic students learn how to recognize mental states.

## 1. Introduction

To the best of our knowledge, Autism Spectrum Condition (ASC) is a neurodevelopmental condition marked by challenges in communication and social interaction [1,2]. These challenges persist throughout life, and despite ongoing learning, difficulties in social and communication skills remain. This is evident even among individuals without cognitive impairment who were diagnosed with Asperger Syndrome (AS) or High-Functioning Autism (HFA) [3,4,5,6]. The conceptualization of autism as a spectrum has revolutionized our understanding of this complex neurodevelopmental condition. This spectrum perspective emerged in the mid-20th century as clinicians and researchers recognized the heterogeneous nature of autism. It has driven research into the genetics, neuroscience, and etiology of autism, aiming to provide more targeted therapies and effective support. A significant shift occurred with the DSM-V [7], which replaced distinct subtypes with a unified diagnosis of Autism Spectrum Disorder (ASD). This change challenges the traditional view of autism as a singular entity, emphasizing personalized approaches to assessment and intervention, and reshaping societal attitudes toward autism. Autism encompasses individuals with a wide range of symptoms, abilities, and challenges under the umbrella term ASC. Despite this diversity, all individuals with ASC share cognitive and behavioral difficulties in communication and social interaction, often accompanied by restricted or repetitive behaviors and interests. People with ASC exhibit behaviors that differ from those of most others, showing limited interest in social stimuli and, depending on the severity of their condition, facing challenges in social situations due to reduced social skills (e.g., language development, gaze perception, and emotion recognition). Moreover, social communication and interaction pose significant challenges, given their avoidance of eye contact, lack of response to name calling, limited facial expression recognition, and difficulty in matching facial expressions with the emotional content of the spoken language.

Humans are inherently social creatures; from birth, we learn to perceive, reproduce, and use social signals to interact with others. Individuals with ASC, however, struggle with social interaction, reflecting a lack of the use of social cues—such as others’ gaze and emotional expressions—which are essential for navigating the social world. The conceptualization of social difficulties in ASC has been widely debated in recent decades. One prominent cognitive theory of autism suggests that these difficulties stem from a deficit in understanding others’ minds, often referred to as the “Theory of Mind” (ToM) [8], “Mind-reading” [9], or “Mentalizing” [10]. This ability to “mentalize” fully emerges around age five, enabling children to understand others’ beliefs and desires and to empathize with others. Individuals with ASC, with limitations in this ability, may find themselves confused by others’ behavior, struggling to grasp the intentions, emotions, and thoughts behind human actions. This may result in varying degrees of “Mind-blindness” [11] or deficits in “Empathizing” [12].

Neuroscientific studies have identified a network underlying these abilities, often referred to as “the social brain” [13]. This network involves the medial, inferior frontal, and superior temporal cortices, along with the amygdala, and processes social stimuli. Perceptual information, in part, arises from regions in the fusiform gyrus and the adjacent inferior occipital gyrus, which are activated in response to faces. Neuroimaging studies of the ToM in ASC have shown reduced activation in these “social brain” areas compared to controls. For instance, an fMRI study revealed that individuals with ASC exhibit less extensive frontal activation and no activation of the amygdala when tasked with inferring others’ mental states and emotions from pictures of eyes (e.g., the “Eyes Task”) [14].

Another prominent cognitive theory of autism is the “Weak Central Coherence” theory (WCC) [5,15]. WCC posits that individuals with ASC have an enhanced focus on details and difficulties integrating information into a coherent whole—they may “miss the forest for the trees”. This theory suggests that individuals with autism struggle to derive meaning from the social world because they perceive it in a fragmented manner, making it difficult to integrate and contextualize details. Social functioning, which requires the rapid integration of context-dependent information, may be significantly impaired under such conditions. Brain function models in autism suggest that WCC reflects a cognitive outcome of altered connectivity between local, “low-level” neural networks, affecting the processing and integration of information. Research indicates that this disruption in low-level visual information processing, such as reduced connectivity between brain regions, may underlie the social challenges faced by individuals with ASC in using social cues to interpret socioemotional contexts [16,17]. Furthermore, a meta-analysis suggests that individuals with ASC show reduced attention to the social aspects of visual scenes compared to control groups [18].

Research suggests that effective emotion recognition relies on integrating multimodal information, such as visual and auditory cues. Both the Theory of Mind (ToM) and Weak Central Coherence (WCC) theory predict difficulties in this domain for individuals with ASC [19]. Studies investigating emotion recognition in computer models designed to mimic neurotypical perception patterns have further demonstrated that employing multimodal information—such as the simultaneous presentation of facial expressions and acoustic cues—enhances the accuracy and robustness of emotion recognition [20]. This evidence underscores the importance of considering multimodal cues for a comprehensive understanding of emotion recognition, particularly for neurotypical individuals who benefit from the integration of multiple sensory inputs to accurately interpret emotional contexts.

However, many traditional assessment tools used to evaluate emotion recognition in individuals with ASC rely primarily on a single modality, often using static images or presenting stimuli through a single sensory channel. A notable example of this is the Reading the Mind in the Eyes Test (RMET), developed by Baron-Cohen in 1997 [21]. This task, designed to assess the ability to infer emotions and mental states through eye gaze, utilizes only static black-and-white photographs of the eye regions. While the RMET is a widely used tool to evaluate the ToM and has been proven to be valuable in distinguishing individuals with ASC from controls, its limited multimodal presentation makes it less ecologically valid and less reflective of real-life social interactions.

To address this gap, researchers have developed more ecologically valid tools like the “Movie for the Assessment of Social Cognition” (MASC), which incorporates short film clips depicting realistic social interactions, requiring participants to infer the characters’ mental states [22]. However, despite its strengths, the MASC has been criticized for its length and the potential difficulty in interpreting some of its items. Other attempts to move beyond traditional tests include the “Awkward Moments Test” (AMT), which uses short video clips taken from advertisements to depict scenarios that might cause embarrassment or social discomfort [23]. Participants are asked to interpret the social dynamics, emotional states, and intentions of the individuals depicted. However, the AMT has been criticized for its lack of ecological validity. The scripted nature of the videos often fails to capture the spontaneity and complexity of real-life social interactions. Thus, while the AMT represents an advancement in integrating social information through video, it does not fully replicate the nuances of everyday social situations. This limitation highlights the need for tools that offer a more realistic and dynamic assessment of emotion recognition, particularly in the context of autism.

Golan and his collaborators [19] developed the “Reading the Mind in Films” (RMF) task, which provides a more comprehensive assessment of emotion recognition compared to traditional methods. The task utilizes short film clips that present diverse social cues within rich contextual scenarios, offering a more realistic and ecologically valid assessment of emotional understanding in daily life. Participants view these clips and are asked to indicate the complex emotions and mental states of the characters depicted, considering not only their facial expressions and body language but also their vocal intonations and the overall context of the scene. This multimodal approach aligns more closely with how emotions are experienced and recognized in everyday interactions.

Despite the RMF’s potential as a valuable tool for assessing complex emotion and mental state recognition, no Italian version is available; in particular, there is no availability of video clips dubbed in Italian. This is a particularly important point since the Italian population is accustomed to enjoying film and video content in Italian since all foreign films are translated and dubbed into Italian. The film clips used in the RMF task, however, are sourced from movies that do not have Italian dubbing, which presents a considerable challenge for using this task in Italy.

This pilot study aims to address the need for an Italian adaptation of the RMF to enhance the ecological validity of emotion and mental state recognition assessments for individuals with and without ASC in Italy. By translating the original scripts and dubbing the original film clips, we aimed at creating an assessment tool that reflects the complexities of social interactions and fills in this critical gap.

## 2. Materials and Methods

### 2.1. Apparatus

After acquiring all necessary materials from the Autism Research Centre—University of Cambridge website www.autismresearchcentre.com (accessed on 3 December 2024), the initial task was to inspect the 22 film clips used in the task. These clips are from English films released between the late 1990s and early 2000s, with only one film (“*L’ospite d’inverno*”) also released in Italy. Due to the lack of Italian dubbing for most clips, with permission, a script was created capturing all actor dialogues, emotions, and contexts, followed by an accurate Italian translation, subsequently reviewed by two native English speakers fluent in Italian.

Professional voice actors and an audio technician were then engaged to assist with dubbing. An expert in audio and video editing was instrumental in recreating the original background sounds and synchronizing the new dubbing with the actors’ lip movements and timing. Voice actors were sourced from the Fiverr platform https://www.fiverr.com/ (accessed on 3 December 2024), where voice actor profiles were meticulously reviewed to find voices matching the original actors’ characteristics and performance requirements. The selected voice actors were briefed and provided with scripts for their performances. The final dubbed audios were sent to the audio–video editing expert who, after extensive collaboration and effort, produced clips that maintained the original timing, emotion, and overall mood.

At the conclusion of the dubbing and editing process, we proceeded to create an Italian version of the video clips to be used to conduct the same experiment designed by Golan et al. [19], administered via the INQUISIT software (version number 6.6.0) [24]. The dubbed video clips were meticulously revised by the same English–Italian speakers to ensure semantic accuracy. Additionally, minor modifications were made to the original English script to incorporate changes necessitated by the translation. Utilizing the original experiment script, available for free on the Autism Research Centre website, we translated all the remaining sections and material of the experiment (i.e., instructions) into Italian. This Italian translated version of the experiment script was presented to a sample of 20 Italian participants to test its correctness.

### 2.2. Participants

Based on the sample analyzed by Golan and colleagues [19] and a sensitivity analysis conducted using G*power software (version number 3.1.9.5) [25], considering an alpha = 0.05; effect size = 0.5, and power = 0.80, the number of participants was determined to be 44 (20 males and 24 females), with an age range between 19 and 39 years (M = 23.4; SD = 3.77). The sensitivity analysis, yielding a *p*-value of 0.40, supported the adequacy of this sample size. Only essential demographic information, specifically gender and age, was collected from participants.

Participants were divided into two groups (22 participants each) and were asked to fill in the Autism Spectrum Quotient (AQ) questionnaire (see Section 2.3) [26]. The neurotypical sample consisted exclusively of students from Milano—Bicocca University attending any of the graduate or post-graduate programs, recruited through the university’s research platform (Sona System; https://www.sona-systems.com/, accessed on 3 December 2024) who had an AQ score lower than 24. Regarding the autistic participants, our sample was a convenience sample consisting of individuals from *Scuola Futuro Lavoro*—a post-diploma vocational training school (SFL; https://scuolafuturolavoro.it/, accessed on 3 December 2024), who had been attending the filmmaking course for a few months and responded to a flyer that was posted throughout the school sometime prior to the study. All of them had been diagnosed with high-functioning autism, with the exception of two participants. Diagnoses were provided by qualified experienced clinicians, according to the criteria of the Diagnostic and Statistical Manual of Mental Disorders [1,7]. As a manipulation check, these individuals completed the AQ questionnaire as well, reporting significantly higher scores than the control group (see Table 1). Only adults with typical cognitive functioning and without significant comorbidities that could affect task performance were included in the study.

### 2.3. Materials

In accordance with Golan et al. [19], the Autism Spectrum Quotient (AQ) [26] and the Reading the Mind in the Eyes Test (RMET) [27] were administered, along with the newly developed Italian version of the Reading the Mind in Films (RMF) task.

The AQ is a self-report questionnaire designed to assess the degree to which an adult from the general population with a normal IQ exhibits traits associated with the autism spectrum. Scores range from 0 to 50, with higher scores indicating a greater presence of autistic traits (≥23). The validated Italian version of the original AQ, was adapted by Liliana Ruta, a specialist in Child Neuropsychiatry at the University of Catania. Ruta’s adaptation [28] ensured that the questionnaire was both linguistically and culturally appropriate for Italian speakers (https://docs.autismresearchcentre.com/tests/AQ_Adult_Italian.pdf accessed on 3 December 2024).

The RMET is a widely recognized psychological assessment tool designed to evaluate the ability to infer emotional and mental states by examining photographs of individuals’ eye regions. The Italian version of the “Reading the Mind in the Eyes Test” was developed by Marcello Vellante and colleagues in 2013 [29].

In our study, we utilized the same video clips originally selected by Golan and colleagues [19], and presented our participants with 22 short film scenes (5–30 s long, M = 14.8, SD = 9.2) characterized for their dramatic content and frequent emotional interactions. The selection criteria for these clips were based on preliminary testing conducted on 15 adults (7 men and 8 women) randomly selected from the general population. Clips were included if the target answer was chosen by at least 50% of participants and if no incorrect answer (foil) was selected by more than 33% of participants. Six items that did not meet these criteria were excluded, resulting in a final task comprising twenty-two validated items, with a score range between 0 and 22. This rigorous selection process ensures that the chosen clips were effective in eliciting clear and accurate interpretations of the complex emotions depicted, aligning with the goals of the study.

The selected scenes featured 14 characters displaying complex emotions and mental states, such as smugness, awkwardness, and concern. Each scene focused on a main character, and participants were required to label the emotional and mental state of the character at the end of each video clip, selecting from a list of four adjectives. Of the four options, only one was correct, while the other three were distractor “foils”.

In Golan and colleagues’ study [19] the foils were chosen to be comparable to the correct answers in terms of verbal complexity. The authors employed an emotion taxonomy developed by Baron-Cohen and colleagues [30]. This taxonomy included 412 emotions and mental states categorized into six developmental levels. Foils were chosen to match or be simpler than the target word in terms of developmental level. While foils corresponded to certain aspects of the emotional content, such as language, they did not match others, such as intonation or context. As in the original study, our participants were provided with a handout containing definitions of all the target and foil words for reference both before and during the task, translated into Italian.

### 2.4. Procedure

The study was carried out in accordance with the guidelines of the Declaration of Helsinki and its later amendments or comparable ethical standards and was approved by the Ethics Committee of the University of Milano-Bicocca. It was conducted over two experimental sessions (T1 and T2), with a minimum interval of one day between them. The sample of the study was composed of 22 individuals with a diagnosis of ASC and 22 neurotypical individuals. Both groups completed the AQ, the RMF task, and the RMET. Given the limited sample size, we conducted exploratory analyses using *t*-tests and Chi-squared tests to examine potential differences between the groups. In T1, participants provided written informed consent before completing a computerized version of the Italian AQ and the RMET. In T2, participants performed the RMF task, followed by a debriefing session where they had the opportunity to ask questions about the tasks. To ensure optimal conditions, participants were seated 60 cm from a computer monitor and wore headphones to minimize external distractions, consistent with the methodology of Golan and colleagues [19]. The video clips were presented to 44 Italian adults (20 males, 24 females; mean age 24.4) on a computer using INQUISIT milliseconds experimental software (version number 6.6.0) [24].

**Figure 1:**
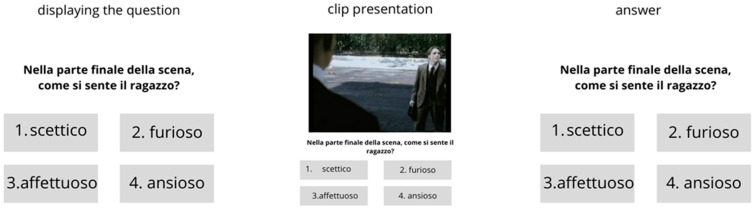
Task sequence in the RMF: participants viewed a question (e.g., “How does the boy feel?”) with four response options, then watched a video clip and selected their answer.

**Figure 2:**
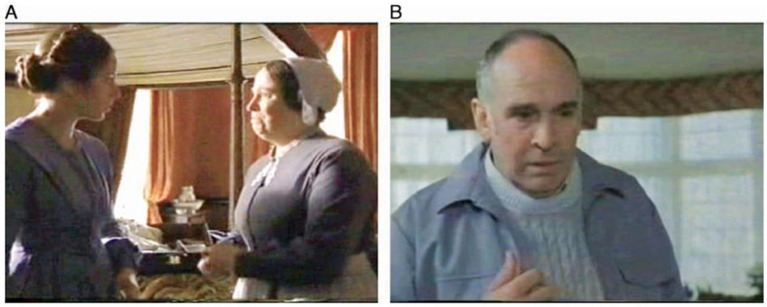
Examples from the RMF task. (**A**) The last frame of the video clip taken from The Turn of the Screw [30] with courtesy of Granada International: participants had to identify the older woman’s emotion (i.e., Overcome). (**B**) The last frame of the video clip taken from Lost for Words [31] with courtesy of ITN Archive: participants had to identify the man’s emotion (i.e., Awkward) as he interrupts a conversation.

Each task began with a question about the scene’s emotional content (e.g., “In the final part of the scene, how does the boy feel?”) followed by four response options, with participants selecting their answer after watching the video clip (Figure 1). Examples of items are shown in Figure 2. For instance, one clip from The Turn of the Screw [31] depicted an older woman emotionally expressing gratitude, with participants asked to identify how she felt from the options of Sociable, Admiring, Overcome, or Liked. Another clip from Lost for Words [32] portrayed a man entering a room full of women, visibly uncomfortable, with participants asked to choose between Ashamed, Unsure, Awkward, or Annoyed. These scenarios highlight the range of social and emotional nuances evaluated in the RMF task.

## 3. Results

### Data Analysis

The statistical analyses were carried out using JAMOVI software (Version number 2.3) [33]. Normality tests were conducted using the Kolmogorov–Smirnov and Shapiro–Wilk methods for the RMET and RMF total scores. For the RMET total, both tests indicated that the data were approximately normally distributed (Kolmogorov–Smirnov: *p* = 0.061; Shapiro–Wilk: *p* = 0.059), with skewness of −0.670 (SE = 0.357) and kurtosis of 0.407 (SE = 0.702). Similarly, the RMF total scores showed no significant deviation from normality (Kolmogorov–Smirnov: *p* = 0.045; Shapiro–Wilk: *p* = 0.244), with skewness of 0.114 (SE = 0.357) and kurtosis of −0.531 (SE = 0.702). These results suggest that the assumption of normality is reasonably met for both measures. A descriptive analysis was also conducted in order to analyze the participants’ responses to the RMF. Specifically, for the 22 target emotions and mental states used in the task, we calculated the percentage of participants who correctly identified them (Table 2). Subsequently, these results were compared with those obtained by Golan and colleagues [19] (Table 3). Task scores were calculated by counting the number of correct answers for each participant. As a manipulation check, participants completed the AQ questionnaire, with the ASC group scoring significantly higher than the control group. Specifically, the ASC group had a mean AQ score of 29.2 (SD = 5.04), while the control group scored a mean of 13.4 (SD = 5.81). An independent sample *t*-test confirmed a statistically significant difference in AQ scores between the groups (t(42) = −9.67, *p* < 0.001), indicating that participants in the ASC group scored significantly higher on the AQ than those in the control group.

In Table 2 and Table 3, we visually compare the participants’ percentages of accuracy for each RMF item with those found by Golan et al. [19]. Focusing on Table 2, which reports our results, we observe differences in the percentages of correct responses between the ASC and the control groups for various emotions. These results indicate that participants in our groups differ, sometimes only slightly, in their ability to recognize and read specific emotions.

Comparing our results with those of Golan et al. [19], intriguing differences in accuracy percentages emerge. For example, in our study, participants in the ASC group show a slightly lower accuracy in recognizing “*Imbarazzato*” (Embarrassed) compared to the control group (36% versus 32%), similar to Golan et al.’s findings (64% versus 68%). Both studies also show that ASC individuals have similar difficulties recognizing certain emotions like “*Amareggiato*” (Bitter), with an accuracy of 18% in both groups across both studies. However, in our study the ASC group exhibited 100% accuracy in recognizing “*Preoccupato*” (Concerned), “*Commosso*” (Overcome), and “*Rassegnato*” (Resigned), unlike in Golan et al.’s study, where they performed worse than the control group for these emotions. For “*Goffo*” (Awkward), our accuracy percentages (18% versus 23%) contrast with Golan’s findings (64% versus 86%), yet still indicate better performance by the control group. For “*Preoccupato*” (Concerned), the accuracy percentages were similar between our study (77% versus 73%) and that of Golan and colleagues (73% versus 73%). These similarities suggest a consistent pattern in the challenges faced by ASC individuals in recognizing specific emotions, as observed in both our research and Golan and colleagues’ study. Additionally, the Italian version of the task used in our study generally showed a wide score range, aligning with the original study’s methodology (see Supplementary materials for the Italian version of the RMF).

**Figure 3:**
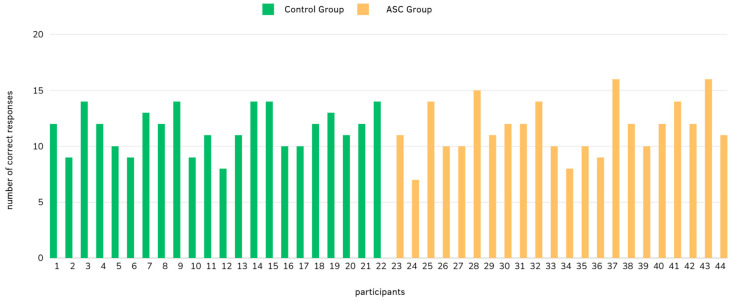
The total number of correct responses (out of 22 times) per each participant (on the *x*-axis) in the RMF task.

The total number of correct responses in the RMF was counted and depicted in the graph below (Figure 3). As evidenced by the graph, the trend of responses is similar across all participants. There is no observable difference between the ASC group (numbers 23 to 44 on the graph) and the control group (1–22) in performance.

**Figure 4:**
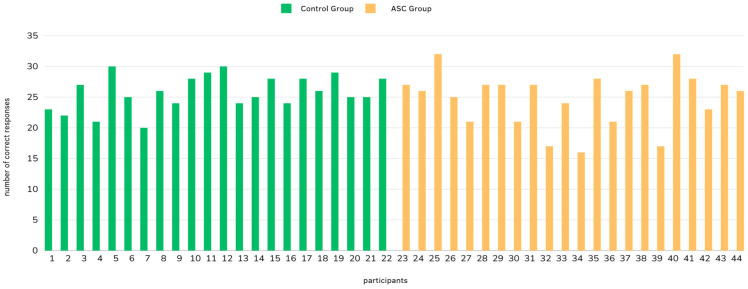
Number of total correct responses (out of 36 items) per each participant (on the *x*-axis) in the RMET.

Subsequently, to further analyze our results, we calculated the percentage of times participants in the control group and ASC groups chose the correct emotion word for each RMF item (see Figure 4). The performance of participants in both the control and ASC groups showed interesting patterns of results across the RMF items. In the initial item, no significant differences were observed between the two groups, with both predominantly selecting the emotion “*Sorpreso*” (Surprised) over the correct response “*Imbarazzato*” (Embarrassed). In the following items, distinct differences in response accuracy and emotion selection became evident. For instance, in the second item, the control group had a higher selection rate for the correct emotion “*Compiaciuto*” (Smug) (44.4%) than the ASC group (31.8%), who more frequently chose “*Subdolo*” (Devious) (45.5%). For some items, both groups showed similar patterns in emotion selection, albeit with slight variations in accuracy. In the seventh item, the ASC group achieved a perfect accuracy rate (100%) in selecting the correct emotion “*Preoccupato*” (Concerned), compared to 90.9% in the control group. Conversely, items like the twelfth displayed similar performance between the two groups, with the correct response “*Pungente*” (Prickly) chosen by approximately half of the participants in both groups. However, certain items showed a higher accuracy rate in the ASC group, such as the ninth item, where the correct emotion “*Commosso*” (Overcome) was consistently chosen by the ASC group (100%) compared to the control group (81.8%). Furthermore, we conducted a Chi-squared test to assess whether there were statistically significant differences in the accuracy of responses between the two groups for each individual item of the RMF task. The analysis revealed a statistically significant difference in the performance of the two groups for item 9 (χ^2^ = 5.641, df = 1, *p* = 0.018). While the difference observed in item 20 did not reach statistical significance (χ^2^ = 3.220, df = 1, *p* = 0.073), the result nonetheless highlights a noticeable divergence in the responses of the two groups to this item. No other significant difference was found for the remaining items.

In summary, variability underscores the nuanced differences in emotional and mental state recognition abilities between the two groups (see Figure 3). Graphs illustrating all the choices made by each group for every task item can be found in the Appendix A.

Moreover, a comparison of performance in the Reading the Mind tasks between our two groups was conducted. Specifically, two independent sample *t*-tests were performed to compare the proportions of correct responses between the ASC and the control groups on the RMF task and the RMET (see Figure 4). For the RMF task, the analysis revealed no statistically significant difference in performance between individuals in the ASC and control groups (t(42) = 0.0800, *p* = 0.937). Similarly, for the Italian version of the “Reading the Mind in the Eyes” task, no statistically significant difference in performance was found between the two groups (t(70) = 0.654, *p* = 0.515).

## 4. Discussion

In this pilot study, which aimed to translate and adapt the RMF task, we found minimal differences in response accuracy between our participants and those in the original study by Golan et al. [19], suggesting that our dubbing and translation efforts were both effective and successful. This alignment highlights the robustness of our careful translation process.

Our findings underscore specific difficulties in recognizing certain emotions among individuals with higher-autistic traits, particularly with emotions like *Goffo* (Awkward), *Lieto* (Pleased), and *Compiaciuto* (Smug), consistent with observations by Golan et al. [19]. These difficulties may stem from the cinematic clips not fully conveying the intended emotions, leading participants to rely on alternative emotional cues. These cues could be influenced by the complex interplay of contextual, performative, verbal, and prosodic elements within the scenes. Moreover, it is noteworthy that, although only a few items of the RMF task were distinguishable between individuals with ASC and individuals with low-autistic traits (i.e. the control group), this is an important result that speaks for the sensitivity of the RMF to test the ability to recognize mental states and emotions, even in this new Italian version.

Although some RMF task scenes may not fully reflect everyday life in Italy, particularly in 2023 (data collection), these cultural and temporal differences did not significantly affect the insights gained from the study. While the rapid pace and certain cultural nuances of the scenes may have added complexity, this did not hinder the test ability to assess emotion recognition in our sample. Nevertheless, to further enhance the ecological relevance of social cognitive tests, future research should consider adapting test materials to better align with contemporary sociocultural contexts. Incorporating scenes that resonate more closely with participants’ daily experiences could improve both the accuracy and applicability of these assessments.

Furthermore, our results indicate no statistically significant differences in performance on the RMET between the ASC and the control groups, whereas it differed only in two items (#9 and #20) out of twenty-two in the RMF task. A possible explanation for this unexpected finding may relate to the unique characteristics of our ASC group, which comprised individuals with substantial exposure to filmmaking, videomaking, and scriptwriting courses at *Scuola Futuro Lavoro*. This specialized training likely enhanced their familiarity with photographs, emotional expressions, and cinematic portrayals, thereby positively influencing their performance on both emotion recognition tasks. Indeed, the absence of significant differences in emotion recognition between the ASC and the control groups may reflect the benefits of consistent exposure to emotional stimuli and training. According to Galván and colleagues [34], the human brain exhibits notable structural and functional plasticity throughout life in response to learning and exposure. In the present study, individuals with ASC engaged in vocational courses that emphasized emotional understanding, likely facilitating improvements in their emotion and mental states recognition skills. Consequently, their increased experience in fields requiring nuanced interpretations of human emotions may have allowed them to perform comparably to the control group, which contrasts with the generally reported lower performance associated with ASC in the literature. This finding highlights the potential for targeted training and exposure to enhance emotion recognition abilities in individuals with ASC, suggesting a valuable avenue for future research and intervention strategies.

Overall patterns of response accuracy were similar between the two groups. While the ASC group generally showed slightly lower accuracy in some emotions, their performance on certain items, such as, for example, recognizing “*Preoccupato*” (Concerned), “*Commosso*” (Overcome), and “*Rassegnato*” (Resigned), was notably high and even surpassed that of the control group. The similarity in performance between the two groups suggests that the processes underlying emotion comprehension may operate comparably, regardless of differences in their levels of autistic traits. Additionally, the consistency of results from our control group with those of the original study as well as the existence of some difference between the ASC and the control groups in performance in the present study reinforces the reliability of our findings and supports our translation and dubbing process of the RMF task.

While we acknowledge certain limitations in our methodology compared to Golan and colleagues [19], such as the lack of formal language comprehension testing, it is worth noting that all students at *Scuola Futuro Lavoro* successfully completed high school, came from Italian families, and were fluent in Italian. Furthermore, we did not pre-test whether the original selection procedure for the 22 clips used in the RMF task would also apply to our sample. Future research should consider these aspects to enhance the adaptation of the RMF task for use in the Italian context.

Nevertheless, our findings reveal an intriguing similarity in performance between both groups (ASC; control) in the administered tests (RMET; RMF). This unexpected result suggests that differences in academic vs. non-academic education are unlikely to account for it. Notably, the non-university group, comprising individuals with ASC, actively participated in film-related vocational courses that involved filmmaking, scriptwriting, and film analysis. This consistent exposure to activities requiring engagement with expressive emotions, facial analysis, and storytelling likely contributed to their proficient performance in emotion and mental state recognition tests. Moreover, these findings imply that active involvement in filmmaking courses may enhance emotional and mental state recognition abilities, providing valuable insights for potential interventions for individuals on the autism spectrum.

A consideration for future research is the absence of a formal IQ measurement in our study. Unlike Golan et al. [19], who used the Wechsler Abbreviated Scale of Intelligence WAIS [35] to assess IQ, we did not administer this test, resulting in a lack of objective IQ measures. Our assumption that participants fell within the normal IQ range was based on the premise that all individuals with autism spectrum conditions (ASC) in our sample were high-functioning and enrolled in a post-diploma school, indicating they likely possess typical cognitive abilities. The alignment of behavioral responses across groups alleviates concerns regarding potential cognitive disparities. Our decision to forgo additional testing was pragmatic, aiming to minimize participant burden. While acknowledging this limitation, we emphasize that the primary focus of the present pilot study was to test the Italian adaptation we made of the RMF task. Future research should systematically explore the role of IQ and the cognitive profiles of individuals with ASC in relation to performance in the RMF task, incorporating formal IQ measurements to provide a more nuanced understanding of cognitive factors influencing emotional and mental state recognition.

## 5. Conclusions

In conclusion, this pilot study effectively validated the reliability and accuracy of the dubbing process, which successfully mirrors the original clips while preserving their emotional content and intentionality. The findings suggest that participation in professional courses requiring extensive exposure to film and video—along with the emotional narratives they depict—may enhance performance in complex emotion and mental state recognition tasks. This potential benefit may stem from the immersive nature of these courses, which engage students within complex emotional scenarios and foster a nuanced understanding of emotional expression.

Future research should explore this hypothesis further, ideally through longitudinal studies that examine performance changes among individuals with autism spectrum conditions (ASC) engaged in similar training programs. Such studies could identify specific elements of film and video exposure that contribute to enhanced emotional recognition skills.

Moreover, expanding the participant pool to include neurotypical individuals and those with difficulties in understanding and expressing emotions (e.g., individuals with alexithymia) could provide valuable insights into the broader applicability of the RMF task. This comparative approach may reveal how different cognitive and emotional profiles affect performance on emotion recognition tasks, potentially informing more targeted interventions and educational strategies. By systematically investigating these directions, future research could make meaningful contributions to our understanding of emotional recognition, improving both diagnostic tools and support strategies for individuals with ASC and related conditions.

## Figures and Tables

**Table 1:** Means, standard deviations, and ranges of AQ, chronological age for the ASC and control groups.

	ASC Group (n = 22)			Control Group (n = 22)		
	Mean	SD	Range	Mean	SD	Range
**AQ**	29.2	5.04	23–42	13.4	5.81	3–22
**Age**	23.3	4.74	19–39	23.5	2.30	20–29

**Table 2:** The table shows the percentage of correct responses in the RMF and presents the Italian translation of the word list from Table 3 by Golan et al. [19].

Emotion/Mental State	ASC Group	Control Group
Seccato	95	82
Goffo	18	23
Sminuito	41	27
Amareggiato	18	18
Preoccupato	100	91
Sconcertato	64	82
Prova antipatia	32	41
Imbarazzato	36	32
Divertito	54	41
Esasperato	82	91
Furibondo	54	68
Commosso	100	82
Lieto	32	36
Pungente	54	50
Riflessivo	50	45
Risentito	41	50
Rassegnato	100	91
Compiaciuto	32	44
Duro	32	27
Afflitto	50	64
Senza pretese	45	36
Preoccupato	77	73

**Table 3:** The table reports the percentage of correct responses in the RMF by Golan et al. [19].

Emotion/Mental State	ASC Group	Control Group
Annoyed	77	73
Awkward	64	86
Belittled	45	68
Bitter	18	18
Concerned	55	77
Disconserted	45	91
Disliking	50	82
Embarrassed	64	68
Enjoying	64	100
Exasperated	64	82
Incensed	68	86
Overcome	59	82
Pleased	77	91
Prickly	36	14
Reflective	50	64
Resentful	36	41
Resigned	59	73
Smug	73	86
Stern	64	55
Trubled	50	59
Unassuming	73	95
Worried	73	73

**Figure A1:**
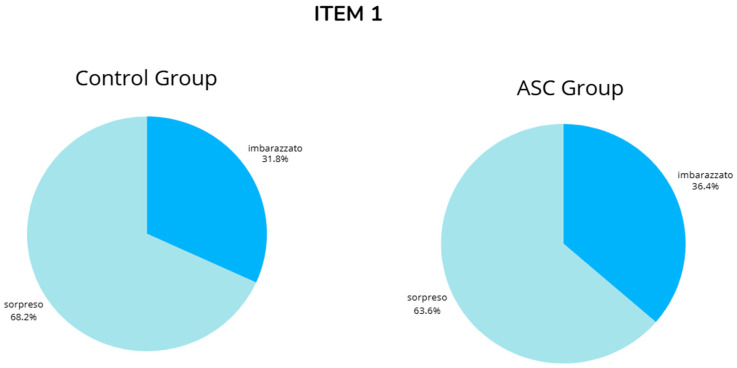
Emotional terms and their frequencies for item 1 of RMF.

**Figure A2:**
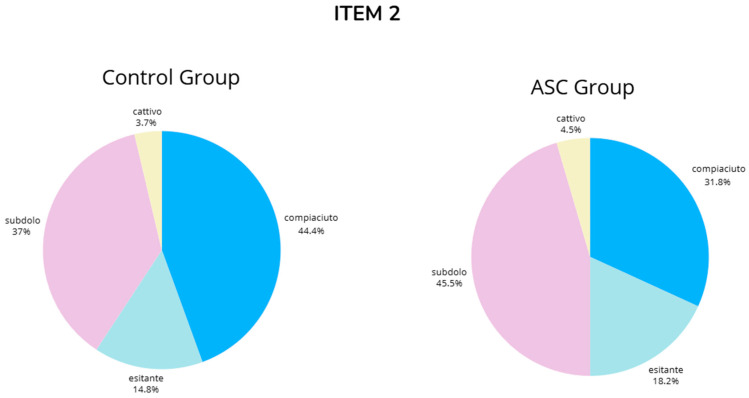
Emotional terms and their frequencies for item 2 of RMF.

**Figure A3:**
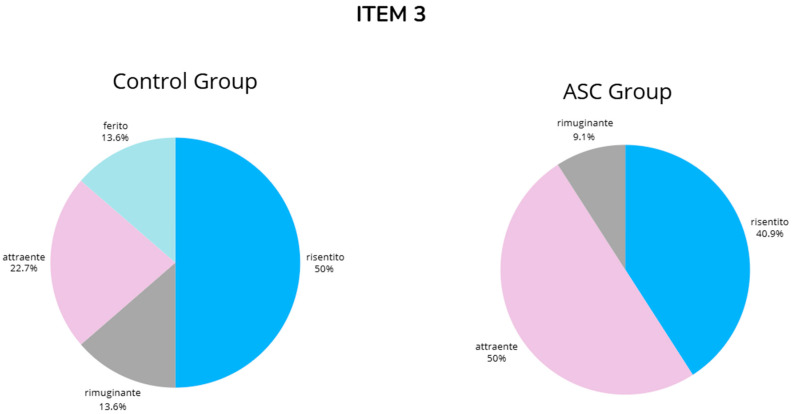
Emotional terms and their frequencies for item 3 of RMF.

**Figure A4:**
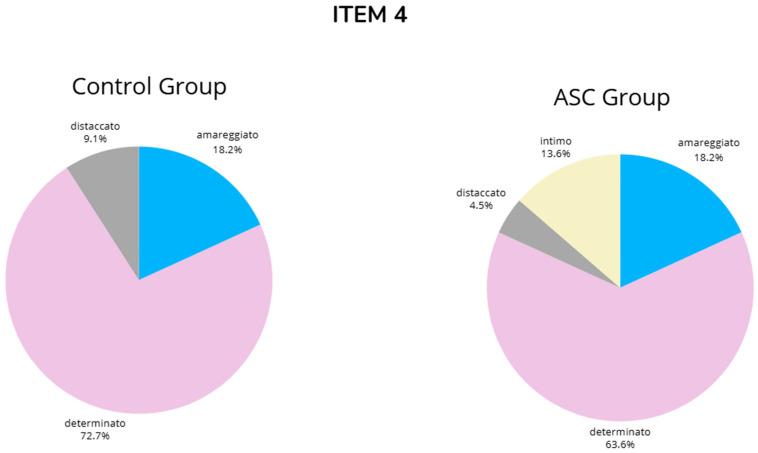
Emotional terms and their frequencies for item 4 of RMF.

**Figure A5:**
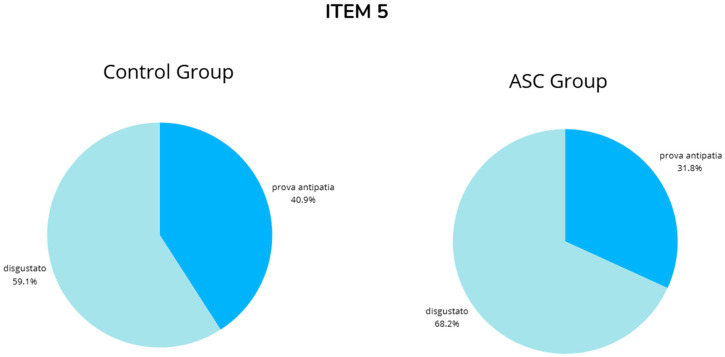
Emotional terms and their frequencies for item 5 of RMF.

**Figure A6:**
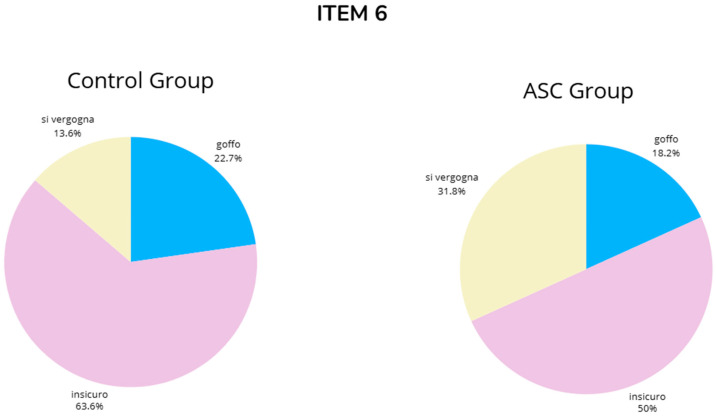
Emotional terms and their frequencies for item 6 of RMF.

**Figure A7:**
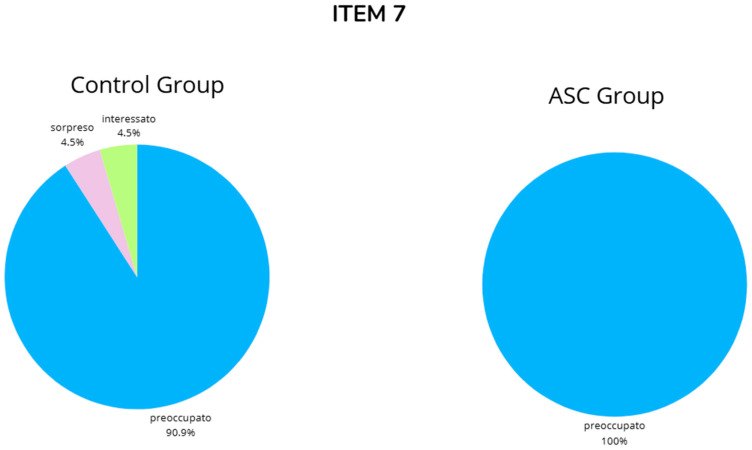
Emotional terms and their frequencies for item 7 of RMF.

**Figure A8:**
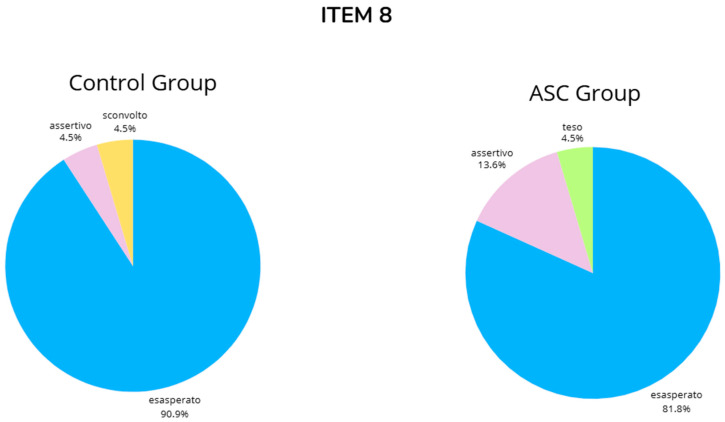
Emotional terms and their frequencies for item 8 of RMF.

**Figure A9:**
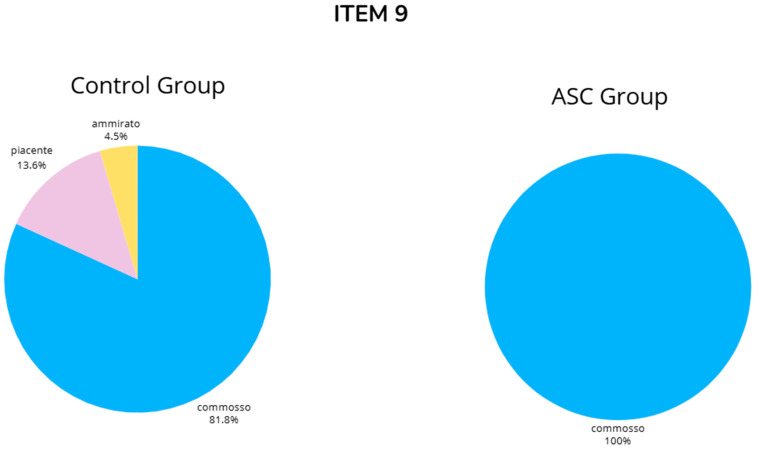
Emotional terms and their frequencies for item 9 of RMF.

**Figure A10:**
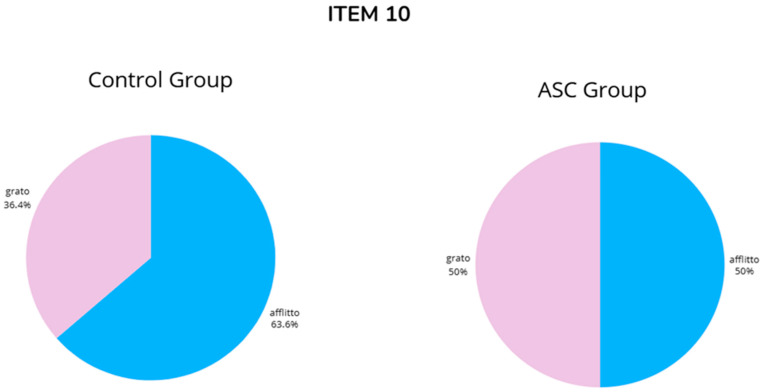
Emotional terms and their frequencies for item 10 of RMF.

**Figure A11:**
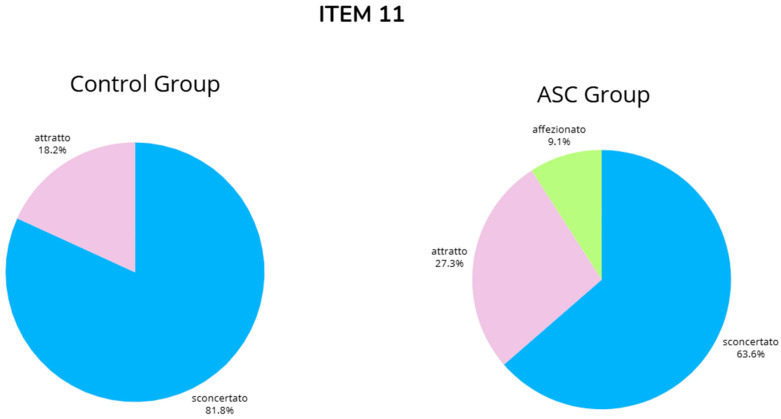
Emotional terms and their frequencies for item 11 of RMF.

**Figure A12:**
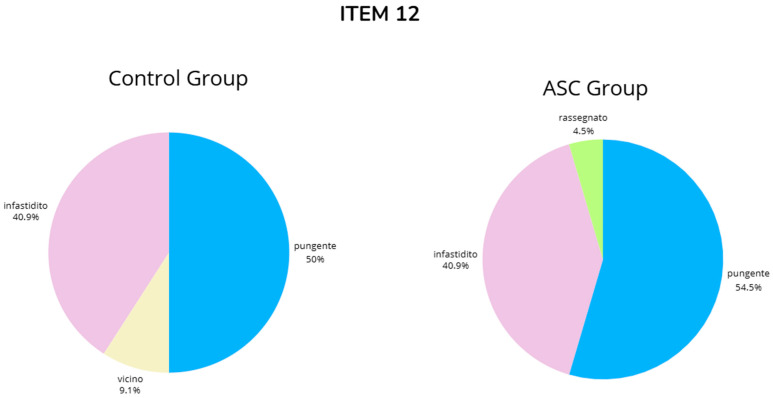
Emotional terms and their frequencies for item 12 of RMF.

**Figure A13:**
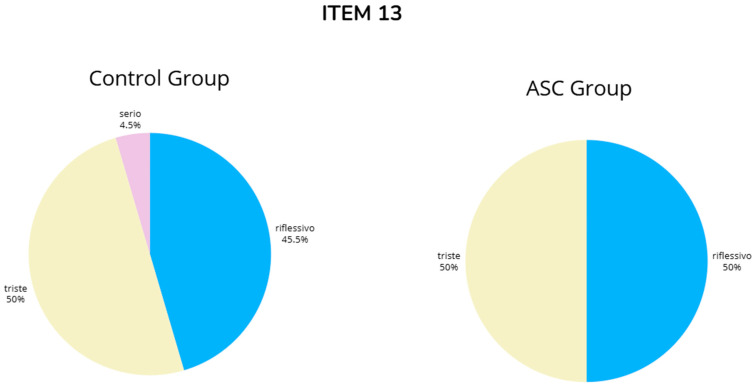
Emotional terms and their frequencies for item 13 of RMF.

**Figure A14:**
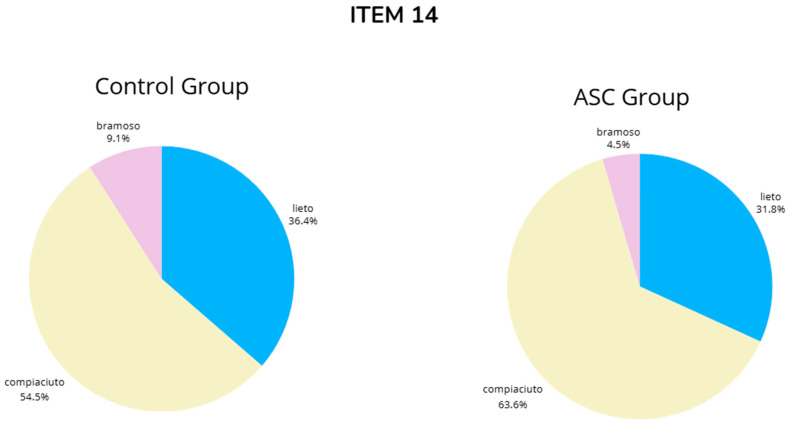
Emotional terms and their frequencies for item 14 of RMF.

**Figure A15:**
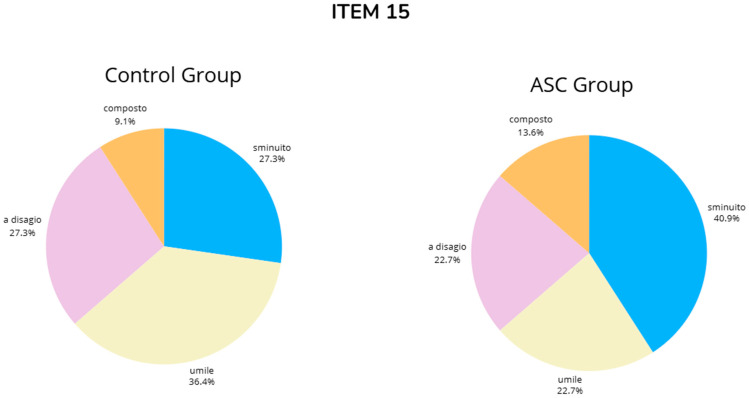
Emotional terms and their frequencies for item 15 of RMF.

**Figure A16:**
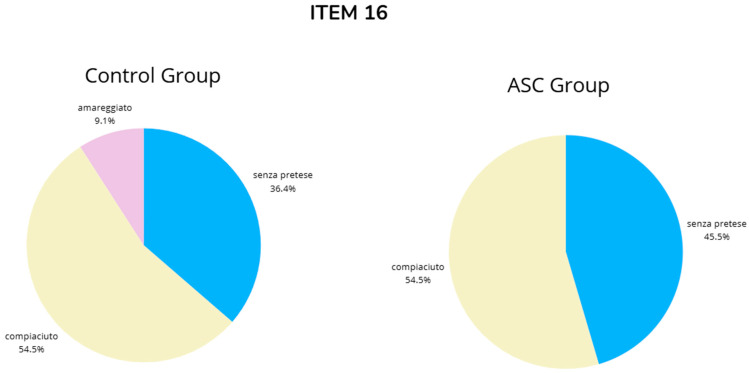
Emotional terms and their frequencies for item 16 of RMF.

**Figure A17:**
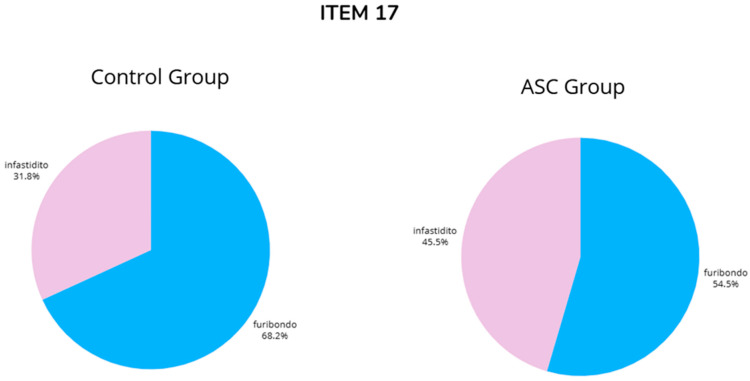
Emotional terms and their frequencies for item 17 of RMF.

**Figure A18:**
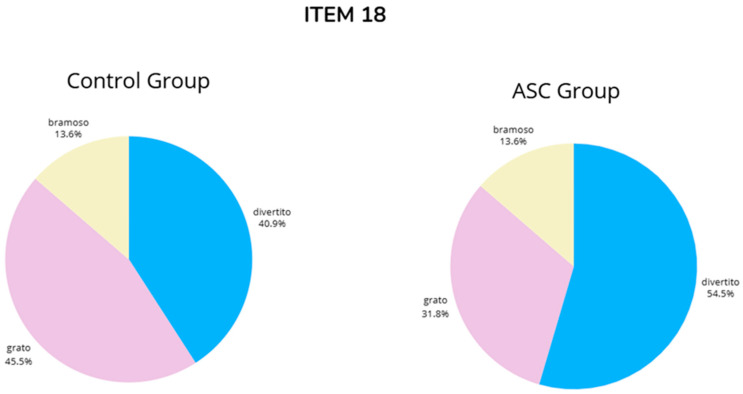
Emotional terms and their frequencies for item 18 of RMF.

**Figure A19:**
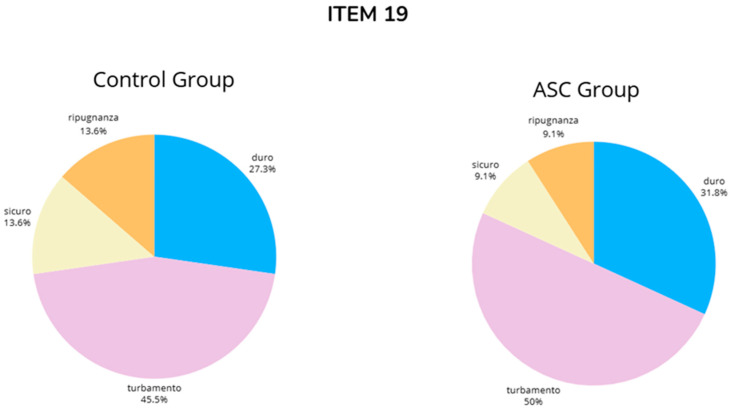
Emotional terms and their frequencies for item 19 of RMF.

**Figure A20:**
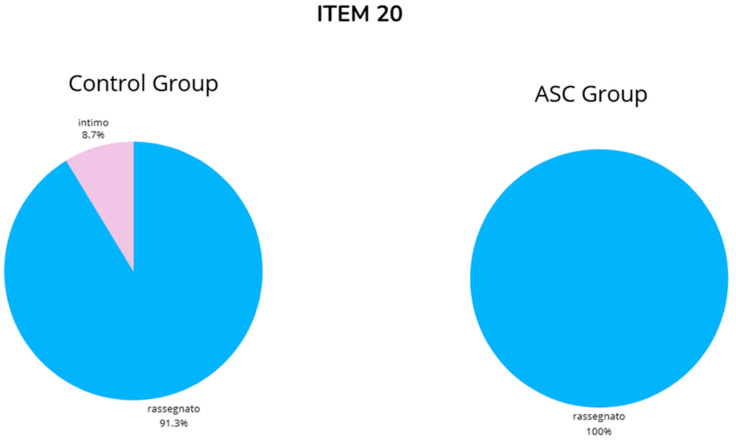
Emotional terms and their frequencies for item 20 of RMF.

**Figure A21:**
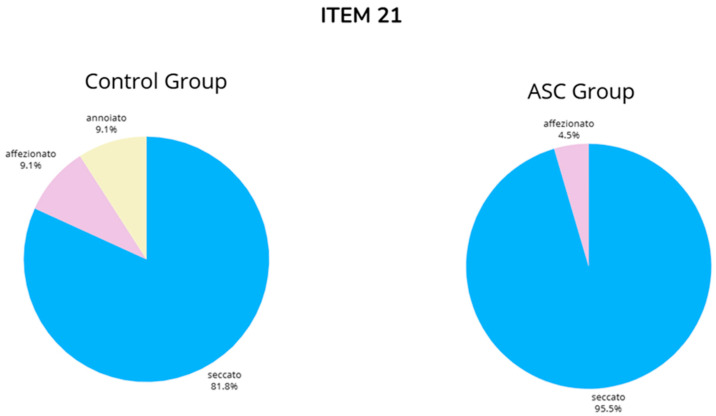
Emotional terms and their frequencies for item 21 of RMF.

**Figure A22:**
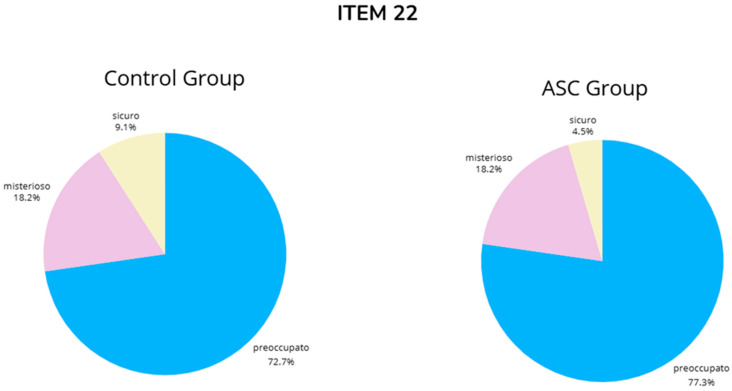
Emotional terms and their frequencies for item 22 of RMF.

## Data Availability

The datasets generated for this study will be deposited on a data repository (OSF; https://osf.io/, accessed on 3 December 2024) and make available on request to monitor the download activity. All the data have been de-identified to preserve the privacy of research participants.

## Supplementary Materials

The Italian version of the task used to support the findings can be accessed at https://www.autismresearchcentre.com/tests/reading-the-mind-in-films-test (accessed on 3 December 2024).

## Appendix A. Emotional Response Frequencies in RMF Items: Comparison Between ASC and Control Groups

The graphs reported below refer to each of the 22 RMF items and display for each emotional response term their response frequencies, both for the controls and the ASC group. The correct response is highlighted in electric blue (see Figure A1, Figure A2, Figure A3, Figure A4, Figure A5, Figure A6, Figure A7, Figure A8, Figure A9, Figure A10, Figure A11, Figure A12, Figure A13, Figure A14, Figure A15, Figure A16, Figure A17, Figure A18, Figure A19, Figure A20, Figure A21 and Figure A22).

Starting from the first item presented in the RMF, neither group exhibited a significant difference in their responses. For both groups, the only other emotion selected besides the correct one “Imbarazzato” (31.8% for the control group versus 36.4% for the ASC group) was “Sorpreso” (68.2% for the control group versus 63.6% for the ASC group). The other potential options were “Infastidito” and “Interessato” (Figure A1).

In the second item presented, besides the correct answer (Compiaciuto), each of the various options was chosen. The correct response was predominantly selected by the control group (44.4%), followed by “Subdolo” (37%), “Esitante” (14.8%), and “Cattivo” (3.7%). The ASC group predominantly linked the character in the clip to “Subdolo” in the majority of instances (45.5%). The accurate emotion (Compiaciuto) was selected merely 31.8% of the time, indicating a notably poorer performance than the control group (Figure A2).

In the third item, notable discrepancies emerged in the responses between the two groups. The control group predominantly opted for the correct response “Risentito” (50%), followed by “Attraente” (22.7%), “Ferito” (13.6%), and “Rimuginante” (13.6%). In contrast, within the control group, 50% of the selections selected “Attrente”, while 40.9% chose the correct option (Risentito), with only a marginal 9.1% opting for “Rimuginante” (Figure A3).

As for the fourth item, for both groups, the most commonly selected emotion word was “Determinato” (72.7% for the control group versus 63.3% for the ASC group), followed as a secondary choice by the correct response “Amareggiato”, with an accuracy percentage of 18.2% for both groups. Within the control group, only one other option, “Distaccato”, was chosen at 9.1%. However, in the ASC group, both “Intimo” (13.6%) and “Distaccato” (4.5%) were also selected (Figure A4).

The performance on the fifth item exhibited a comparable outcome across the two groups; both groups favored the option “Disgusted” (59.1% for the control group versus 68.2% for the ASC group). The ASC group selected the correct response “Aversion” a fewer number of times than the control group, indicating a better performance by the control group (40.9% for the control group versus 31.8% for the ASC group). The other potential options were “Insicuro” and “Nervoso” (Figure A5).

Both groups in the sixth item more frequently chose the emotion “Insicuro” with a rate of 63.6% for the control group and 50% for the ASC group. The correct option “Goffo” was selected only 22.7% of the time by the control group and 18.2% by the ASC group. The other option chosen by both groups was “Si vergogna”, accounting for 13.6% for the control group and 31.8% for the ASC group. As evident from these results, both groups predominantly associated the mood of the main character in the clip with an emotion different from the correct one. However, the group with lower-autistic traits performed comparatively better than the group with higher-autistic traits (Figure A6).

For the seventh item, the ASC group consistently chose the correct emotion 100% of the time, showcasing exceptional accuracy and precision. Conversely, the control group opted for the correct response “Preoccupato” 90.9% of the time, with “Interessato” and “Sorpreso” each at 4.5% (Figure A7).

The performance in the eighth item slightly varied between the two groups. Indeed, both predominantly selected the correct option “Esasperato” (90.9% for the control group versus 81.8% for the ASC group). However, there is a notable difference in the remaining emotions chosen. Both groups opted for “Assertivo”, with the higher-autistic-traits group selecting it more frequently (13.6%), followed by “Teso” (4.5%). Conversely, the lower-autistic-traits group chose “Assertivo” and “Sconvolto” with equal frequency, each at 4.5% (Figure A8).

In the ninth item, the ASC group consistently chose the correct emotion (Commosso) in every instance, while the control group selected “Commosso” only 81.8% of the time, opting for “Piacente” in 13.6% of cases and “Ammirato” in 4.5% of cases (Figure A9).

In the tenth item, the control group outperformed the group with higher-autistic traits, choosing the correct response “Afflitto” 63% of the time, followed by “Grato” at 36.6%. Conversely, the other group selected “Afflitto” and “Grato” 50% of the time, respectively (Figure A10).

Analyzing the performance of the two groups in the eleventh item, the control group exhibited a superior performance, preferring the correct option (Sconcertato) 81.8% of the time, followed by a solitary other emotion (Attratto), chosen 18.2% of the time. The ASC group selected the correct option only 63.6% of the time, with a considerable number of participants choosing “Attratto” (27.3%) and “Affezionato” (9.1%) (Figure A11).

In the twelfth item, the correct response “Pungente” was chosen by the group with lower-autistic traits 50% of the time, followed by “Infastidito” (40.9%) and “Vicino” (9.1%). Meanwhile, in the ASC group, the correct response was chosen 54.5% of the time, also followed by “Infastidito” at a frequency of 40.9%, and “Rassegnato” in 4.5% of cases (Figure A12).

Observing the responses to the thirteenth item, for both groups, the answer “Triste” was selected 50% of the time, followed by the correct response “Riflessivo” with a frequency of 45.5% for the ASC group and 50% for the control group. In the control group, another emotion was chosen (Serio) in 4.5% of cases (Figure A13).

In the fourteenth item, the emotion “Compiaciuto” was chosen most often by both groups, with a frequency of 54.5% among control participants and 63.6% among ASC participants. The correct response “Lieto” was selected in 36.4% of cases by the control group, followed by “Bramoso” at 9.1%. For the ASC group, the correct response was chosen in 31.8% of cases, also followed by “Bramoso” at 4.5% (Figure A14).

In the fifteenth item, both groups selected all four options, but with different frequencies. In the control group, “Umile” was the most chosen (36.4%), followed by the correct option “Sminuito” (27.3%), then “A disagio” (27.3%), and “Composed” (9.1%). In the ASC group, “Sminuito” was the predominant choice (40.9%), followed by “Umile” and “A disagio” (22.7% each), and “Composed” (13.6%) (Figure A15).

As for the sixteenth item, the frequencies at which the correct emotion (Senza pretese) was chosen was 36.4% in the control group and 45.5% in the ASC group. Conversely, the control group predominantly selected “Compiaciuto” in 54.5% of cases, and also chose “Amareggiato” (9.1%). (Figure A16).

In the seventeenth item, “Furibondo” (the correct response) was the most chosen response by both groups, selected 68.2% of the time by the control group and 54.5% by the ASC group. Participants also chose “Infastidito”, selected 31.8% of the time by the ASC group and 45.5% by the control group (Figure A17).

In the eighteenth item, the correct response “Divertito” was chosen by the control group only 40.9% of the time. Surprisingly, the most frequently chosen emotion was “Grato” at a rate of 45.5%, while “Bramoso” was also selected, albeit not often (13.6%). In the ASC group, “Divertito” was accurately chosen in 54.5% of instances. “Grato” was selected only 31.8% of the time, and “Bramoso” in 13.6% of the cases (Figure A18).

Regarding the performance in the nineteenth item, the most chosen option by both groups was “Turbamento”, selected 45.5% of the time by the control group and 50% by the ASC group. The correct emotion (Duro) was selected in 27.3% of cases by the control group and in 31.8% of cases by participants with ASC. “Ripugnanza” and “Sicuro” were chosen 13.6% of the time by the control group, and 9.1% of the time by the ASC group (Figure A19).

In the twentieth item, the ASC group unanimously chose the correct response “Rassegnato”, demonstrating a ceiling performance. In contrast, the control group chose the correct emotion in 91.3% of instances. The alternative option selected was “Intimo”, noted in 8.7% of cases (Figure A20).

An enhanced performance by the ASC group is discernible in the twenty-first item, where they consistently chose the accurate option (Seccato) 95.5% of the time and selected “Affezionato” in only 4.5% of the instances. In contrast, the control group selected the correct response 81.8% of the time, followed by “Annoiato” and “Affezionato” (9.1% each) (Figure A21).

To conclude, in the final item, the correct response (Preoccupato) was chosen 72.7% of the time by the control group and 77.3% by the ASC group. Other options selected were “Misterioso” (18.2% for both groups) and “Sicuro” (9.1% for control group and 4.5% for high-autistic-traits group) (Figure A22).

## References

[1] American Psychiatric Association (2000). Diagnostic and Statistical Manual of Mental Disorders.

[2] World Health Organization (1994). ICD-10*International Classification of Diseases.

[3] Attwood T. (1998). Asperger’s Syndrome: A Guide for Parents and Professionals.

[4] Baron-Cohen S., Tager-Flusberg H., Cohen D.J. (2000). Understanding Other Minds: Perspectives from Developmental Cognitive Neuroscience.

[5] Frith U. (1989). Autism: Explaining the Enigma.

[6] Hobson R.P. (1993). Autism and the Development of Mind.

[7] American Psychiatric Association (2013). Diagnostic and Statistical Manual of Mental Disorders.

[8] Astington J.W., Harris P.L., Olson D.R. (1988). Developing Theories of Mind.

[9] Wellman H.M. (1992). The Child’s Theory of Mind.

[10] Frith C., Frith U. (2005). Theory of mind. Curr. Biol..

[11] Baron-Cohen S. (1995). Mindblindness: An Essay on Autism and Theory of Mind.

[12] Baron-Cohen S., Wheelwright S., Lawson J., Griffin R., Hill J., Goswami U. (2002). The exact mind: Empathizing and systemizing in autism spectrum conditions. Blackwell Handbook of Childhood Cognitive Development.

[13] Brothers L., Ring B. (1992). A neuroethological framework for the representation of minds. J. Cogn. Neurosci..

[14] Baron-Cohen S., Ring H.A., Wheelwright S., Bullmore E.T., Brammer M.J., Simmons A., Williams S.C. (1999). Social intelligence in the normal and autistic brain: An fMRI study. Eur. J. Neurosci..

[15] Happe F.G. (1999). Autism: Cognitive deficit or cognitive style?. Trends Cogn. Sci..

[16] Belmonte M.K., Allen G., Beckel-Mitchener A., Boulanger L.M., Carper R.A., Webb S.J. (2004). Autism and abnormal development of brain connectivity. J. Neurosci..

[17] Critchley H.D., Daly E.M., Bullmore E.T., Williams S.C., Van Amelsvoort T., Robertson D.M., Rowe A., Phillips M., McAlonan G., Howlin P. (2000). The functional neuroanatomy of social behavior: Changes in cerebral blood flow when people with autistic disorder process facial expressions. Brain.

[18] Chita-Tegmark M. (2016). Social attention in ASD: A review and meta-analysis of eye-tracking studies. Res. Dev. Disabil..

[19] Golan O., Baron-Cohen S., Hill J.J., Golan Y. (2006). The “reading the mind in films” task: Complex emotion recognition in adults with and without autism spectrum conditions. Soc. Neurosci..

[20] Busso C., Deng Z., Yildirim S., Narayanan S. Analysis of emotion recognition using facial expressions, speech and multimodal information. Proceedings of the 6th International Conference on Multimodal Interfaces, ICMI 2004.

[21] Baron-Cohen S., Jolliffe T., Mortimore C., Robertson M. (1997). Another advanced test of theory of mind: Evidence from very high functioning adults with autism or Asperger syndrome. J. Child Psychol. Psychiatry.

[22] Dziobek I., Fleck S., Kalbe E., Rogers K., Hassenstab J., Brand M., Kessler J., Woike J.K., Wolf O.T., Convit A. (2006). Introducing MASC: A movie for the assessment of social cognition. J. Autism Dev. Disord..

[23] Heavey L., Phillips W., Baron-Cohen S., Rutter M. (2000). The Awkward Moments Test: A naturalistic measure of social understanding in autism. J. Autism Dev. Disord..

[24] (2016). Inquisit 5 [Windows 11]. https://www.millisecond.com.

[25] Faul F., Erdfelder E., Lang A.-G., Buchner A. (2007). G*power 3: A flexible statistical power analysis program for the social, behavioral, and biomedical sciences. Behav. Res. Methods.

[26] Baron-Cohen S., Wheelwright S., Skinner R., Martin J., Clubley E. (2001). The Autism-Spectrum Quotient (AQ): Evidence from Asperger syndrome/ high-functioning autism, males and females, scien tists and mathematicians. J. Autism Dev. Disord..

[27] Baron-Cohen S., Wheelwright S., Jolliffe T. (1997). Is there a ‘‘language of the eyes’’? Evi dence from normal adults, and adults with autism or Asperger syndrome. Vis. Cogn..

[28] Ruta L., Mazzone D., Mazzone L., Wheelwright S., Baron-Cohen S. (2012). The Autism-Spectrum Quotient—Italian version: A cross-cultural confirmation of the broader autism phenotype. J. Autism Dev. Disord..

[29] Vellante M., Baron-Cohen S., Melis M., Marrone M., Petretto D.R., Masala C., Preti A. (2013). The “Reading the Mind in the Eyes” test: Systematic review of psychometric properties and a validation study in Italy. Cogn. Neuropsychol..

[30] Baron-Cohen S., Wheelwright S. (2004). The empathy quotient: An investigation of adults with Asperger syndrome or high functioning autism, and normal sex differences. J. Autism Dev. Disord..

[31] Bolt B. (1999). The Turn of the Screw [Film].

[32] Bell A.J.W. (1999). Lost for Words [Film].

[33] (2023). Jamovi Project. Jamovi (Version 2.3) [Windows 11]. https://www.jamovi.org.

[34] Galván A. (2010). Neural plasticity of development and learning. Hum. Brain Mapp..

[35] Wechsler D. (1999). Wechsler Abbreviated Scale of Intelligence.

